# ELOVL gene family plays a virtual role in response to breeding selection and lipid deposition in different tissues in chicken (Gallus gallus)

**DOI:** 10.1186/s12864-022-08932-8

**Published:** 2022-10-17

**Authors:** Dandan Wang, Xinyan Li, Panpan Zhang, Yuzhu Cao, Ke Zhang, Panpan Qin, Yulong Guo, Zhuanjian Li, Yadong Tian, Xiangtao Kang, Xiaojun Liu, Hong Li

**Affiliations:** 1 College of Animal Science and Technology, Henan Agricultural University, Zhengzhou, 450046 China; 2 Henan Institute of Veterinary Drug and Feed Control, Zhengzhou, 450002 China; 3 Henan Key Laboratory for Innovation and Utilization of Chicken Germplasm Resources, Zhengzhou, 450046 China; 4 International Joint Research Laboratory for Poultry Breeding of Henan, Zhengzhou, 450046 China

**Keywords:** *ELOVL* gene family, SNP, Fat deposition, Expression regulation, Chicken

## Abstract

**Background:**

Elongases of very long chain fatty acids (ELOVLs), a family of first rate-limiting enzymes in the synthesis of long-chain fatty acids, play an essential role in the biosynthesis of complex lipids. Disrupting any of *ELOVLs* affects normal growth and development in mammals. Genetic variations in *ELOVLs* are associated with backfat or intramuscular fatty acid composition in livestock. However, the effects of *ELOVL* gene family on breeding selection and lipid deposition in different tissues are still unknown in chickens.

**Results:**

Genetic variation patterns and genetic associations analysis showed that the genetic variations of *ELOVL* genes were contributed to breeding selection of commercial varieties in chicken, and 14 SNPs in *ELOVL*2-6 were associated with body weight, carcass or fat deposition traits. Especially, one SNP rs17631638T > C in the promoter of *ELOVL3* was associated with intramuscular fat content (IMF), and its allele frequency was significantly higher in native and layer breeds compared to that in commercial broiler breeds. Quantitative real-time PCR (qRT-PCR) determined that the *ELOVL3* expressions in pectoralis were affected by the genotypes of rs17631638T > C. In addition, the transcription levels of *ELOVL* genes except *ELOVL5* were regulated by estrogen in chicken liver and hypothalamus with different regulatory pathways. The expression levels of *ELOVL*1-6 in hypothalamus, liver, abdominal fat and pectoralis were correlated with abdominal fat weight, abdominal fat percentage, liver lipid content and IMF. Noteworthily, expression of *ELOVL3* in pectoralis was highly positively correlated with IMF and glycerophospholipid molecules, including phosphatidyl choline, phosphatidyl ethanolamine, phosphatidyl glycerol and phospholipids inositol, rich in ω-3 and ω-6 long-chain unsaturated fatty acids, suggesting *ELOVL3* could contribute to intramuscular fat deposition by increasing the proportion of long-chain unsaturated glycerophospholipid molecules in pectoralis.

**Conclusions:**

In summary, we demonstrated the genetic contribution of *ELOVL* gene family to breeding selection for specialized varieties, and revealed the expression regulation of *ELOVL* genes and their potential roles in regulating lipid deposition in different tissues. This study provides new insights into understanding the functions of *ELOVL* family on avian growth and lipid deposition in different tissues and the genetic variation in *ELOVL3* may aid the marker-assisted selection of meat quality in chicken.

**Supplementary Information:**

The online version contains supplementary material available at 10.1186/s12864-022-08932-8.

## Background

Poultry meat is an important component of healthy human diets. Over the past several decades, more and more attentions have been paid to improving quality in addition to continuing to pursue for quantity in poultry meat production industry. In chicken, a number of candidate genes and genetic variations that potentially affect growth and meat quality have been identified by different genetic analysis approaches in various chicken populations [1–7]. And the responses to selection for growth rate and meat quality in broiler chickens were accelerated with the rapid development of genome sequencing and chip technology [8–10]. However, the mechanisms underlying the growth and meat quality still remain poorly understood.

Genetic variation is one of the main factors driving phenotypic changes. Domestication and artificial selection in a given species altered the genome variation and thus driven the diversification and specialization of phenotypes among varieties [11]. For example, there are abundant varieties and categories of chicken, including the original red jungle fowl, different indigenous varieties, and specialized commercial broilers and layers. A range of genetic variants and functional genes have been identified that were associated with production phenotypes, such as body weight [3] and fat deposition [6] in chicken. Elongase of very long chain fatty acids (ELOVLs) from ELOVL1 to ELOVL7 are responsible for synthesizing long chain fatty acid (LCFA, > 16 carbons) and also the first rate-limiting enzyme in the carbon chain elongation reaction of LCFAs, which play an essential role in the synthesis of complex lipids that are an integral part of adipose deposition in different tissues [12]. The *ELOVL* genes are widely expressed in multiple tissues [13, 14] and involved in a variety of physiological and pathological regulatory processes in mammals [15]. Disrupting any of the *ELOVLs* blocked the in vivo synthesis of LCFAs, which significantly affected normal growth [16] and development [17] in mouse models, and increased the risk of fatty liver [18], insulin resistance and cardiovascular disease [19]. These previous evidences showed that the *ELOVL* genes family played an indispensable role in various biological functions by coordinating the synthesis and metabolism of FAs and the composition of FAs in different tissues. What's more, numerous reports have shown that single nucleotide polymorphisms (SNPs) occurred in the *ELOVL* genes in humans were associated with some disorders such as the high frequency deafness [20], the alterations in obesity related blood lipid parameters and body mass index [21], and several distinct tissue-specific blindness, aggressive juvenile-onset retinal degeneration or early-childhood seizures [22]. In domestic animals, genetic variations in *ELOVL5* and *ELOVL7* are significantly associated with backfat [23] and intramuscular fatty acid composition in porcine [24], while *ELOVL6* gene is identified as a candidate gene controlling the homeostasis in fatty acid composition in porcine, subsequently, for governing important economic traits such as meat quality [25]. However, to the best of our knowledge, the patterns of genetic variation in *ELOVL* genes of different chicken varieties, and their potential correlation with growth, lipid deposition and meat quality phenotypes have not yet been systematically studied.

Chicken fat deposition mainly includes liver fat deposition, abdominal fat deposition and intramuscular fat deposition. However, the role of *ELOVL* gene family in lipid deposition of different tissues are still unclear, and it is also unclear that which fatty acids or lipid molecules are affected by *ELOVLs* expression. In addition, estrogen is a vital hormone for sexual maturity and the development of the female reproductive system [26], and it often involves in the regulation of lipid metabolism in chicken [27]. Our previous study revealed that in long chain acyl-CoA synthetases (*ACSL*) gene family, the transcription activities of *ACSL1*, *ACSL3* and *ACSL4* were silenced, while *ACSL6* was activated by estrogen in chickens [28]. As the first rate-limiting enzyme of synthesizing LCFA, it is not clear whether the expressions of *ELOVL* gene family are regulated by estrogen in chicken.

In the present study, we systematically characterized the genetic variation patterns of *ELOVL* genes in different chicken varieties, and inferred whether such patterns are influenced by breeding selection. We investigated the genetic association between SNPs in *ELOVL* genes and chicken production phenotypes, analyzed the expression regulation of *ELOVL* genes in vivo, explored the correlations between the expression levels of *ELOVL* genes and lipid deposition in different tissues and lipid molecular composition in intramuscular fat in chicken. Our findings bring new insight into understanding the function roles of *ELOVL* gene family in breeding selection and lipid deposition in different tissues in chicken.

## Results

### Genetic variation pattern of *ELOVL* genes are in response to breeding selection in chicken

**Fig. 1:**
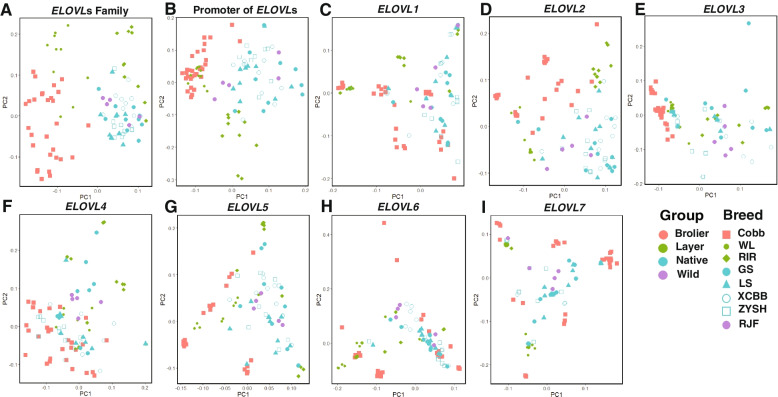
Principal component analysis plots based on SNPs mapping to: (**A**) the whole-genome of 7 *ELOVL* genes, (**B**) the 2 kb promoter region of *ELOVL*s, (**C**) *ELOVL1*, (**D**) *ELOVL2*, (**E**) *ELOVL3*, (**F**) *ELOVL4*, (**G**) *ELOVL5*, (**H**) *ELOVL6*, (**I**) *ELOVL7*, respectively. Cobb, Cobb broiler; WL, White Leghorn layer; RIR, Rhode Island Red layer; GS, Gushi chicken; LS, Lushi chicken; XCBB, Xichuan Black Bone chicken; ZYSH, Zhengyang San Huang chicken; RJF, Red jungle fowl

Based on whole-genome SNPs in the *ELOVL* genes (distributing in 2 kb promoters, 5’UTRs, exons, introns and 3’UTRs) across multiple chicken breeds, we identified 5731 SNPs mapping to 7 *ELOVL* genes and observed that commercial broilers or layers separated from wild and native chicken via PCA analysis (Fig. 1A). The genetic differentiation between commercial breeds and wild or native breeds was also evident when we analyzed population structure based on the promoter region SNPs of *ELOVL*s (Fig. 1B). This implies that the genetic variation of *ELOVL*s in the specialized commercial chickens might be influenced by intensive artificial selection during breeding. Especially, *ELOVL3* gene variation were shaped by selection for meat-production (Cobb) (Fig. 1E), and *ELOVL2*, *ELOVL5* and *ELOVL7* gene variations were shaped by selection for meat-production (Cobb) and egg-production (WL and RIR) during modern breeding, whereas the large genetic diversity among commercial individuals was still apparent (Fig. 1D, G and I). For the *ELOVL1*, *ELOVL4* and *ELOVL6* genes, there was no significant separation of commercial breeds from wild and native chicken (Fig. 1C, F and H). However, PCA analysis showed high genetic diversity in native chickens (Fig. 1A-I), hinting at the potential for genetic directed selection in the further.

### Genotypes of SNPs in *ELOVL* genes are associated with growth, carcass and meat quality traits in chicken

**Fig. 2:**
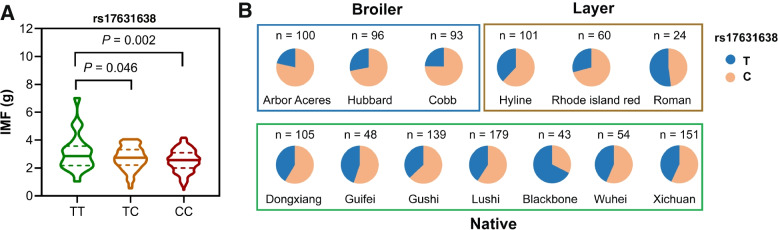
Genotype association of SNP rs17631638T > C in the promoter of *ELOVL3* with intramuscular fat content (IMF) in Gushi-Anka F2 Chickens (**A**), and the allelic frequency in different breeds (**B**)

A total of 51 SNPs distributed in the aforementioned genomic regions of *ELOVL* gene family except *ELOVL1* and *ELOVL7*, among which *ELOVL2* contained 9 SNPs, *ELOVL3* contained 2 SNPs, *ELOVL4* contained 11 SNPs, *ELOVL5* contained 11 SNPs, and *ELOVL6* contained 18 SNPs. Association analysis showed that 25 of the 51 SNPs were significantly associated with growth, carcass or meat quality traits in Gushi × Anka F2 chicken population (Additional file 1: Table S1). And 14 of the 25 SNPs in *ELOVL* gene family were confirmed the significant associations with traits in the same population (Additional file 2: Table S2), combining with the linkage disequilibrium analysis (Additional file 3: Figure S1). Interestingly, we identified an association between SNP rs17631638T > C in the promoter of *ELOVL3* and IMF at 12 weeks (*P* < 0.05). Genotype TT was beneficial to the increase of IMF (Fig. 2A). The allele frequency of T in rs17631638 was evidently lower than that of C in commercial broiler breeds, while its allele frequency was significantly increased in native and layer breeds (Fig. 2B). These results that the SNPs in *ELOVLs* affected chicken growth, carcass and meat quality traits to varying degrees, further supported the deduction that breeding-selection contributed to variety-specific phenotypes.

### Genotypes of rs17631638 in promoter of *ELOVL3* affect gene expression Level

**Fig. 3:**
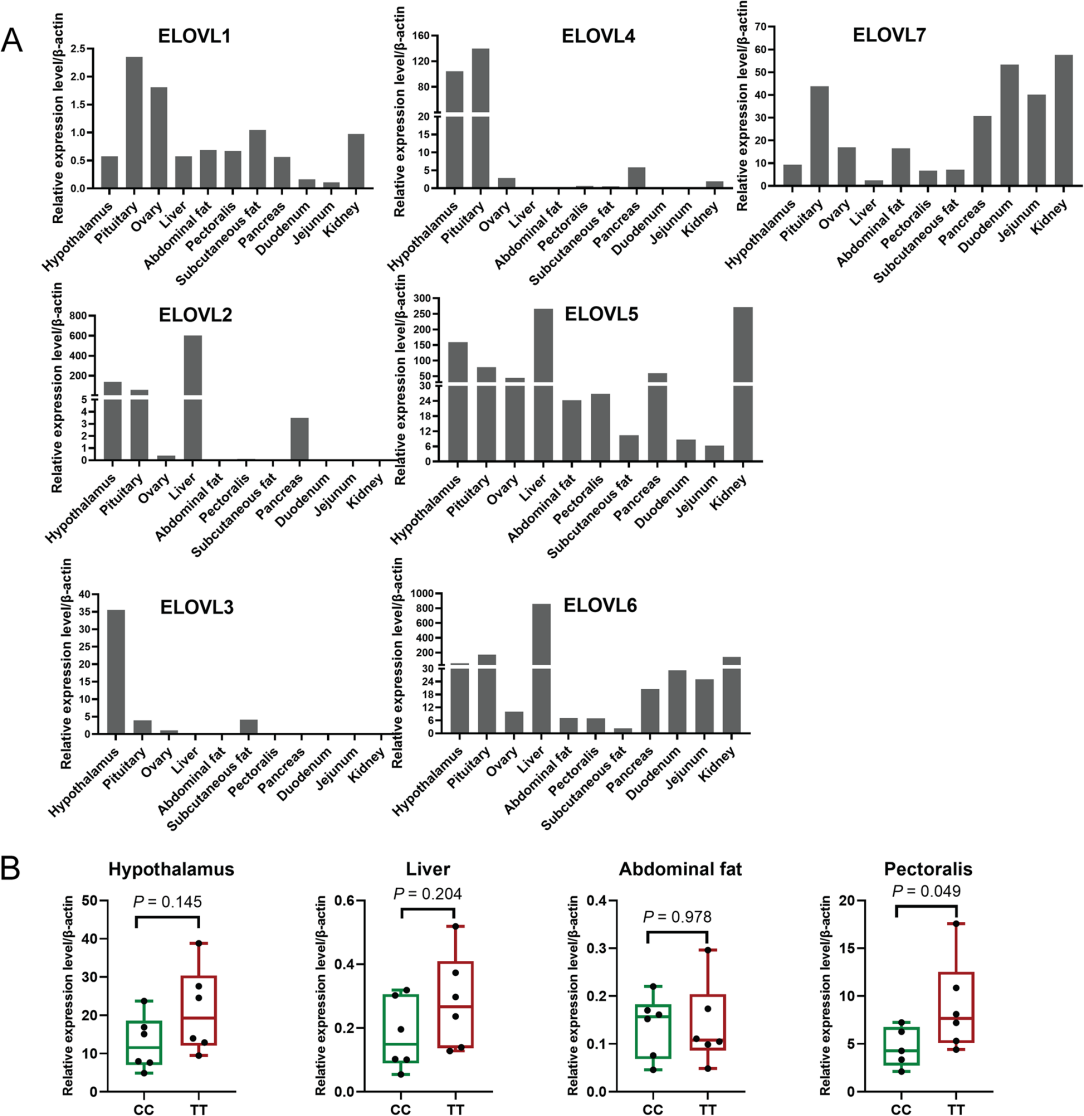
Genotypes of rs17631638 affect the expression Level of *ELOVL3.* **A** Tissue expression profile of *ELOVL* gene family. **B** Comparison of mRNA expression of ELOVL3 between TT and CC genotype of rs17631638 in hypothalamus, liver, abdominal fat and pectorals (*n* = 6). Results are expressed as the mean ± SEM

The expression profiles of *ELOVL*s were first analyzed in 11 tissues including hypothalamus, pituitary, ovary, liver, abdominal fat, pectoralis, subcutaneous fat, pancreas, duodenum, jejunum, and kidney from 43-week-old chickens. The results showed that the expression patterns of the ELOVLs were different between each other (Fig. 3A). Further, we compared the expression levels of *ELOVL3* in chicken hypothalamus, liver, abdominal fat and pectoral tissues among individuals with different genotypes by qRT-PCR. The results showed that *ELOVL3* expression was significantly higher in pectoralis of individuals with TT genotype compared with those who hold CC genotype in rs17631638 (*P* < 0.05; Fig. 3B).

### Expression of *ELOVL* genes are regulated by estrogen in chicken

**Fig. 4:**
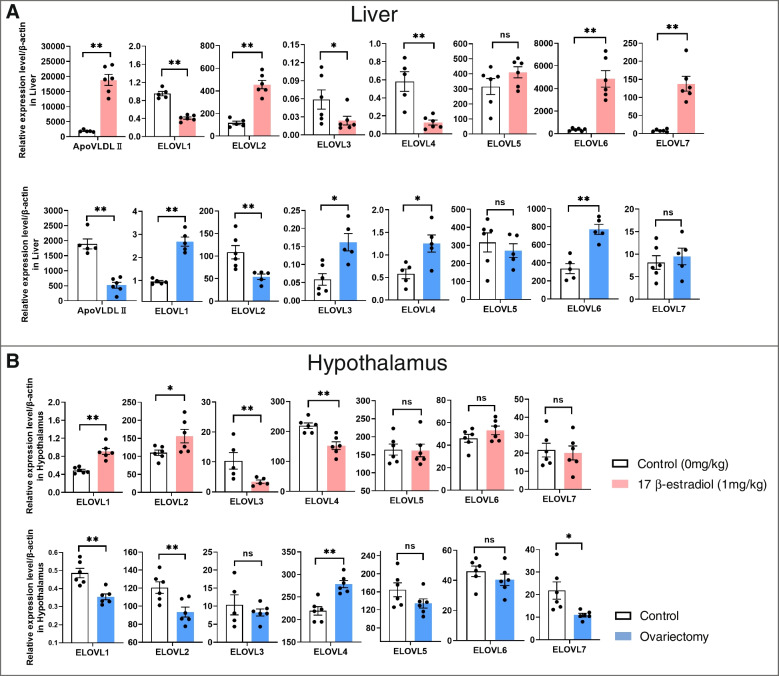
Expression of *ELOVL* genes are regulated by estrogen in chicken. **A** Expression analyses of *ELOVL* genes under 17β-estradiol treatment and ovariectomy in liver of 10-week chicken. **B** Expression analyses of *ELOVL* genes under 17β-estradiol treatment and ovariectomy in hypothalamus of 10-week chicken. The mRNA levels of genes were normalized to *β-actin*. Results are presented as the mean ± SEM and the normalized expression values for all individuals (*n* = 6). Significant difference: * *P* < 0.05, ** *P* < 0.01. ns: no difference

*ELOVL*s expression responding to estrogen was investigated by using estrogen treated and ovariectomized chickens in vivo. The *ELOVL*s expression levels were detected in liver and hypothalamus after 17 β-estradiol II (*ApoVLDL II*), an estrogen treatment and ovariectomy in 10-week-old chickens. Hepatic apovitellenin very-low-density lipoprotein response gene, was significantly up-regulated in chicken treated with 17β-estradiol, and was significantly down-regulated with ovariectomy, indicating that estrogen treatment and ovariectomy are effective in chicken (*P* < 0.05, Fig. 4A).

In liver, the expression levels of *ELOVL1*, *ELOVL3* and *ELOVL4* showed a significant decrease with 17 β-estradiol treatment (*P* < 0.05), but a significant increase with ovariectomy (*P* < 0.05). In contrast, the expression level of *ELOVL2* was significantly elevated under the exposure of estrogen (*P* < 0.05), but was significantly decreased with the elimination of estrogen (*P* < 0.05). The expression levels of *ELOVL6* and *ELOVL7* were significantly increased after treatment with 17 β-estradiol (*P* < 0.05), but not changed in the birds with ovariectomy. Moreover, no estrogen response for expression of *ELOVL5* was observed (Fig. 4A).

In hypothalamus, the expression of *ELOVL1* was significantly upregulated in the 17 β-estradiol treatment group (*P* < 0.05), and was significantly downregulated in the ovariectomized group (*P* < 0.05). The expression patterns of *ELOVL2*, *ELOVL3* and *ELOVL4* were similar to that in liver. The expressions of *ELOVL5*, *ELOVL6* and *ELOVL7* hardly changed in 17 β-estradiol treated and ovariectomized hypothalamus, except *ELOVL7*, which was significantly reduced in the hypothalamus after ovariectomy (*P* < 0.05). Collectively, only *ELOVL5* expression was not regulated by estrogen in chicken (Fig. 4B).

Estrogen response elements prediction showed that a putative ERα binding site occurs in the promoter regions of the six *ELOVL* genes except *ELOVL1*, a putative ERβ site occurs in the promoter regions of the five *ELOVL* genes except *ELOVL4* and *ELOVL6*, and a putative AP-1 site occurs in the promoter region of the *ELOVL3*, *ELOVL4* and *ELOVL7* genes (Additional file 4: Table S3). These results suggested that the expression of *ELOVL*1-6 genes are regulated by estrogen through these potential estrogen response elements in chicken.

### Expression levels of *ELOVL* genes are correlated with lipid deposition in different tissues

**Fig. 5:**
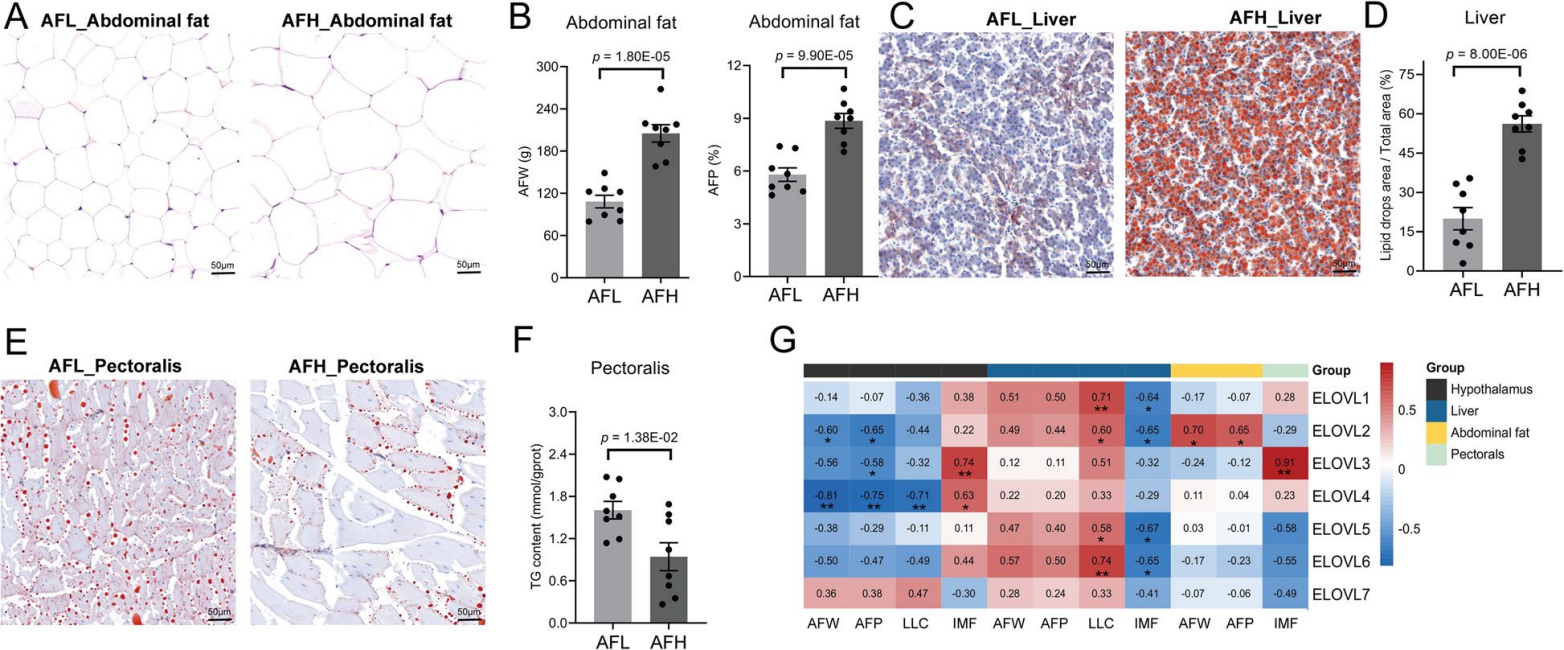
Differences of lipid phenotypes and their correlation with multitissue *ELOVL*s expression in Gushi chickens at 43 weeks. **A** Differences in cell number and lipid droplet area of abdominal fat in the low (AFL) and high abdominal fat group (AFH) of Gushi chickens determined by hematoxylin–eosin (H&E) staining. Scale bar: 50 μm (40X). **B** Abdominal fat weight (AFW) and abdominal fat percentage (AFP) in AFL and AFH group (*n* = 8 for each group). **C** The difference in lipid droplet accumulation of liver in AFL and AFH group determined by oil red O staining. **D** Quantification of Liver lipid content (LLC) in AFL and AFH group. **E** The difference in lipid droplet accumulation of pectoralis in AFL and AFH group determined by oil red O staining. **F** Pectoral TG content in AFL and AFH group. Pectoral TG content represents intramuscular fat content (IMF). **G** Correlation analysis of *ELOVL*s expression in hypothalamus, abdominal fat, liver and pectorals with AFW, AFP, LLC and IMF. The numbers in the box represent Pearson correlation coefficients. Significant difference: * *P* < 0.05, ** *P* < 0.01

Further, we explored the correlations between *ELOVLs* expression and lipid deposition in the tissues including abdominal fat, liver, and pectoralis. A total of 16 43-week-old Gushi hens were divided into high abdominal fat group (AFH, *n* = 8) and low abdominal fat group (AFL, *n* = 8) according to AFW and AFP (Fig. 5A). Remarkably increased the diameter, but decreased the number of abdominal fat cells were observed in AFH group in comparison with those in AFL group (Fig. 5B). Simultaneously, the conspicuously superior liver lipid content (LLC) was uncovered in the AFH group compared to those in the AFL group in liver (Fig. 5C and D). However, intramuscular fat content (IMF) in pectorals in AFH group was significantly lower than that in AFL group (*P* < 0.05, Fig. 5E and F).

The expression levels of *ELOVL* genes were quantified in the hypothalamus, liver, abdominal fat and pectoralis in AFH and AFL by qRT-PCR. The *ELOVL1* and *ELOVL2* in liver, *ELOVL3* in hypothalamus and pectorals, *ELOVL4* in hypothalamus, and *ELOVL5* and *ELOVL6* in liver and pectorals were found to be significantly differentially expressed between AFH and AFL (*P* < 0.05, Additional file 5: Figure S2). Furthermore, we observed the correlations between expressions of *ELOVLs* in multi-tissue and lipid deposition related phenotypes including AFW, AFP, LLC and IMF. The expression levels of *ELOVL2*, *ELOVL3* and *ELOVL4* in hypothalamus were significantly negatively correlated with AFW and AFP, and positively correlated with IMF (*P* < 0.05, Fig. 5G), the expression levels of *ELOVL1*, *ELOVL2*, *ELOVL5* and *ELOVL6* in liver were significantly positively correlated with LLC, and negatively correlated with IMF to different degree (*P* < 0.05, Fig. 5G), and the expression levels of *ELOVL2* in abdominal fat were significantly positively correlated with AFW and AFP (*P* < 0.05, Fig. 5G). Especially, there existed an extremely high positive correlation between expression levels of *ELOVL3* and IMF in pectoralis (*R* = 0.91, *P* < 0.01).

## *ELOVL3* expression contributes to the long chain unsaturated glycerophospholipids deposition in intramuscular fat in chicken

**Fig. 6:**
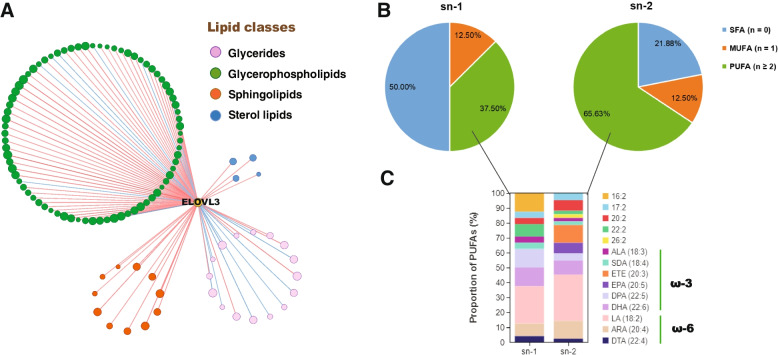
*ELOVL3* expression contributes to the long chain unsaturated glycerophospholipids deposition in intramuscular fat of Gushi chicken. **A** Lipid molecules in intramuscular fat significantly correlated with *ELOVL3* expression in pectoralis of Gushi chicken. The dots represent lipid molecules. Node size represents significant correlation degree, and the larger the node, the higher the correlation degree. The lines between the dots represent Pearson correlations. The red line is positive, and the blue line is negative. Other information is listed on the legend. **B** Proportion of different types of fatty acids at sn-1 and sn-2 positions of the positively correlated glycerophospholipid molecules. SFA (*n* = 0) indicates saturated fatty acids, MUFA (*n* = 1) indicates monounsaturated fatty acids, and PUFA (*n *≥ 2) indicates polyunsaturated fatty acids. **C** Proportion of different types of MUFAs at sn-1 and sn-2 positions in (**B**)

To further investigate which types of lipids and fatty acids are affected by expression of *ELOVL3* gene in pectoralis, we evaluated the association between the expression levels of *ELOVL*3 and multiple lipid molecules in pectoralis of Gushi hens at 43 weeks. The results showed that *ELOVL3* expression level was significantly correlated with 107 lipid molecules, which could be classified into four types including sterol lipids, sphingolipids, glycerophospholipids and glycerides (Additional file 6: Table S4, Fig. 6A), with the most abundance of glycerophospholipids (76/107) (Fig. 6A). In more details, *ELOVL3* expression was mainly positively correlated with 64 lipid molecules in glycerophospholipids, most of which belonged to phosphatidyl choline (PC), phosphatidyl ethanolamine (PE), phosphatidyl glycerol (PG) and phospholipids inositol (PI) (Additional file 6: Table S4). Among the 64 positively correlated lipid molecules, the proportion of unsaturated FAs in sn-1 positions was 50.0%, in sn-2 positions was 78.13% (Fig. 6B). And these polyunsaturated glycerophospholipid molecules were rich in essential ω-3 and ω-6 fatty acids (Fig. 6C). These results suggested that *ELOVL3* expression contributes to the long chain unsaturated glycerophospholipids deposition in chicken intramuscular fat.

## Discussion

Increasing the growth rate, and improving health and meat quality of broilers, especially native chickens are an important long-term breeding goal. ELOVLs, a family of enzymes that catalyze the rate-limiting condensation reaction of LCFA elongation cycle, regulate lipid biosynthesis and fatty acid metabolism [15]. Previous studies revealed that the backfat or intramuscular fatty acid composition were associated with SNPs in the *ELOVL5* [23], *ELOVL6* [25] and *ELOVL7* [24] in porcine. These indicated that *ELOVL* genes family played a vital role in fat deposition, fatty acid composition and meat quality. However, the contribution of the *ELOVL* gene family to the selection of growth and meat quality traits, as well as lipid deposition, remains unclear.

Domestic chickens include native varieties and specialized commercial broiler and layer breeds that have evolved genetic adaptations to environment and have been subjected to strong human-driven selection leading to phenotypic diversity in morphology, physiology and behaviour [11]. In the last 10 years, many studies have explored genetic mechanisms that genetic variations shape phenotypic diversity by genomic comparison studies [3]. PCA analysis revealed the existence of a detectable genetic differentiation among wild, native and commercial chicken populations, with the native populations showing increased levels of diversity compared to the wild or commercial breeds [29]. In our study, the genetic differentiations between the commercial broilers and layers and the wild and native chickens were clearly detected when the whole-genome SNP dataset or the promoter region SNP dataset in the *ELOVL* genes were considered. The intensive artificial selection during breeding altered the growth rate of commercial broilers with changes in abdominal fat deposition and IMF, and the reproductive performance and body fat metabolism of commercial layers [29]. Our results implied that the genetic variations of *ELOVL* genes contributed to phenotypic changes in commercial broilers or layers under the intensive artificial selection. When only the SNP dataset in a single gene of *ELOVLs* was considered, we found that the population differentiation degree between commercial breeds and the wild and native chickens reduced, indicating that these genes acted together to response to the divergent selection. The significant association of SNPs in *ELOVL* genes with growth, carcass or meat quality traits further supported the above findings that the genetic variations of *ELOVL* genes contributed to the variety-specific phenotypic shaping in chicken.

Estrogen plays an important role in transcriptional regulation of genes related to lipid metabolism in poultry, especially in female birds [27]. Estrogen is produced by ovarian follicles with varies in the laying cycle, and has diverse effects in both the brain and the periphery [30]. The 17β-estradiol, the most active estrogen, exerts its effects through the classical nuclear estrogen receptors, but it can also signal through cell surface receptor, G-protein coupled estrogen receptor (GPER, known as GPR30) which is expressed in multiple tissues including liver, hypothalamus and other tissues [31, 32]. The 17β-estradiol treated and ovariectomized experiments revealed that estrogen could inhibit the transcription of *ELOVL1*, *ELOVL3* and *ELOVL4*, on the contrary, promote the transcription of *ELOVL2*, *ELOVL6* and *ELOVL7*, but without effect on that of *ELOVL5* in liver. In hypothalamus, there is a similar expression pattern to that in the liver for *ELOVL2*, *ELOVL3* and *ELOVL4* under estrogen treatment. However, expression of *ELOVL1* was contrary to its pattern in the liver, while expression of *ELOVL5*, *ELOVL6* and *ELOVL7* showed no response to estrogen in hypothalamus. Estrogen response element prediction showed that there were one or more putative ERα, ERβ, or AP-1 binding sites existed in the promoter regions of the seven *ELOVL* genes. Previous studies have shown that crosstalk between the GPR30 and the classical ERs, ERα and ERβ modulates signaling [33], and GPR30 activation may antagonize ERα or ERβ-mediated effects either by suppression of ERα or ERβ expression or their downstream signaling pathways [34]. Since *ELOVL1* only possessed ERβ binding site in its promoter, but had different responses to estrogen in the liver and hypothalamus, we speculated that *ELOVL1* gene could be regulated by estrogen via the crosstalk between the GPR30 and ERβ in liver or hypothalamus. Previous researches showed that the expression of *ELOVL2* was robustly up-regulated upon estradiol stimulation in human breast cancer cell line which expresses ERα but not ERβ, and was almost fully eliminated by knock-down of ERα [35]. In our study, *ELOVL2* only possessed a ERα binding site in its promoter, and was up-regulated by estrogen in the liver and hypothalamus of chicken, implying that estrogen could activate the expression of *ELOVL2* via ERα. Both *ELOVL3* and *ELOVL4* genes possessed the ERE binding sites and AP1 element in their promoters, and were down-regulated by estrogen in both liver and hypothalamus, suggesting that estrogen inhibitory effect of these two genes may be mediated by a classical ERE pathway and a nonclassical AP1 pathway. Despite the presence of multiple ERα and ERβ binding sites in the promoter region of *ELOVL5*, its expression was not affected by estrogen in both liver and hypothalamus, implying that *ELOVL5* gene was not a target of estrogen with inactive receptor binding sites. However, it could be targeted by estrogen-mediated other factors such as miRNA, lncRNA, circRNA and transcription factors. For example, our previous studies showed that estrogen downregulated miR-218-5p thereby augmenting the expression of its target gene *ELOVL5* to enhance hepatic synthesis of LCPUFA [36]. As for *ELOVL6* and *ELOVL7* genes, their expressions were enhanced by estrogen in liver, with one ERα binding site found in *ELOVL6* promoter and multiple ERE binding sites and AP1 element in *ELOVL7* promoter, while the expressions of *ELOVL6* and *ELOVL7* were not decreased with the treatment of ovariectomy, suggesting that *ELOVL6* could be regulated by estrogen via the crosstalk between the ERα and cell surface receptor GPR30, transcription factors or non-coding RNA in liver, as well as *ELOVL7* via the crosstalk between nuclear estrogen receptors and other factors in liver. Our previous study indicated that estrogen abolished the repression role of gga-miR-221-5p on ELOVL6 expression to promote lipid synthesis in chicken liver, supported the regulatory role of estrogen and non-coding RNA interaction against *ELOVL6* [37]. The expression of *ELOVL6* and *ELOVL7* in hypothalamus showed no estrogen response, indicating that the two genes have different estrogen regulation patterns between in liver and hypothalamus tissues.

More importantly, we confirmed the correlations of multi-tissue *ELOVLs* expressions with lipid deposition in liver, abdominal fat and pectoralis of chicken. Systems biology research proposed that complex phenotypic traits were the result of systematic regulation involving multiple tissues and numerous molecular pathways [38]. Here, four tissues including hypothalamus, liver, abdominal fat, and pectoralis were used to analyze the expression of *ELOVL* genes and lipid deposition phenotypes. The hypothalamus was the major regulatory center for energy homeostasis, controlling food intake, energy expenditure, body weight and fatty deposits via affecting the anabolic or catabolic regulation pathways [39]. The liver is the main site of de novo lipogenesis whereas adipocyte serves as the storage site for lipids in birds, and hepatic lipogenesis contributes 80 to 85% of the fatty acids stored in adipose tissue [40]. The intramuscular fat deposition is an important index affecting meat tenderness and taste. Correlation analysis confirmed that the expressions of *ELOVL2*, *ELOVL3* and *ELOVL4* in hypothalamus were significantly negatively correlated with AFW and AFP, and positively correlated with IMF, while the expressions of *ELOVL1*, *ELOVL2*, *ELOVL5* and *ELOVL6* in liver were significantly negatively correlated with IMF, and positively correlated with LLC with different degree, indicating that the high expressions of *ELOVL1*, *ELOVL2*, *ELOVL5*, and *ELOVL6* genes in liver potentially increase liver lipid accumulation and promote abdominal fat deposition to some extent, but not conducive to intramuscular fat deposition in the late growth periods. In addition, the expressions of *ELOVL2* in abdominal fat was significantly positively correlated with AFW and AFP, suggesting the increase of *ELOVL2* expression level in abdominal fat possibly aggravates the accumulation of abdominal fat. Especially, there existed an extremely high positive correlation between *ELOVL3* expression in pectoralis and IMF, implying that *ELOVL3* may be a key gene involved in the positive regulation of intramuscular fat deposition in chicken. Further analysis showed that ELOVL3 expression was mainly positively correlated with 64 glycerophospholipid molecules, most of which belonged to PC, PE, PG and PI. And the sn-2 positions of these lipid molecules were replaced by a large number of ω-3 or ω-6 long-chain polyunsaturated fatty acids. These results suggested that *ELOVL3* could contribute to intramuscular fat deposition by increasing the proportion of the long chain unsaturated glycerophospholipids in chicken. In addition, ω-3 fatty acids, contains eicosapentaenoic acid (EPA), docosapentaenoic acid (DPA) and docosahexaenoic acid (DHA), which are essential precursors of endogenous anti-inflammatory lipid mediators, and increasing the proportion of these glycerophospholipid molecules are also conducive to human health [41]. Therefore, the SNPs that associated with intramuscular fat content on *ELOVL3* could be used as a molecular marker to assist selection for improvement of meat quality in chicken.

## Conclusion

In summary, we demonstrated for the first time that the genetic variations of *ELOVL* genes were responded to the divergent selection for specialized commercial varieties in chicken, and confirmed the associations of SNPs in *ELOVL*2-6 with body weight, carcass or fat deposition traits. As the first rate-limiting enzyme in the carbon chain elongation reaction of LCFAs, the transcription levels of *ELOVL*s except *ELOVL5* were regulated by estrogen with different regulatory pathways. The expressions of *ELOVL*1-6 in multi-tissue were associated with fat deposition in liver, abdominal fat or pectoralis of chicken, among which *ELOVL3* could contribute to intramuscular fat deposition by increasing the proportion of glycerophospholipid molecules rich in long-chain unsaturated fatty acids in pectoralis of chicken. Our findings shedding light on deeper understanding of their genetic and biological functions in avian growth, lipid or fatty acid metabolism.

## Materials and methods

### Characterization of single nucleotide polymorphism (SNP) in *ELOVL* gene family in broilers, layers, native and wild chicken breeds

The whole-genome sequences (WGS) of 95 chickens belonging 4 different types including broiler breed (Cobb, *N* = 30), layer breeds (White Leghorn (WL) and Rhode Island Red (RIR), *N* = 20), native breeds (Gushi (GS), Lushi (LS), Xichuan Black Bone (XCBB) and Zhengyang San Huang (ZYSH) chickens, *N *= 40) and a wild breed (Red jungle fowl (RJF), *N* = 5) were used to detect SNPs in the *ELOVL* gene family. The WGS of Cobb was obtained from China Agricultural University, the WGS of WL, RIR, and RJF were retrieved from published dataset (Additional file 7: Table S5) [42], and the WGS of 4 native breeds was obtained from our previous sequencing data. The detailed information of genomic SNPs in *ELOVL* gene family is available in Additional file 8: Table S6. Principal component analysis (PCA) were used to identify of genetic variation patterns of *ELOVL* gene family in the above multiple chicken breeds.

### Experimental birds and sample preparation

A total of 734 birds from the Gushi × Anka F2 chicken resource population were used for association analysis between genotypes and phenotypes. The Gushi × Anka F2 population were obtained from an F1 generation constructed via reciprocal crossing between Gushi chickens (a slow-growing Chinese native chicken) and Anka broilers (a fast-growing broiler) as described previously [43]. All chickens were kept in the same environmental conditions and had free access to feed and water. Phenotypes used in this study included growth, carcass and meat quality traits, and the measurement methods have been detailed by Han et al. (2010). Growth traits consisted of body weight (BW) from birth to 12 weeks (BW0, BW2, BW4, BW6, BW8, BW10 and BW12). Carcass traits at 12 weeks included carcass weight (CW), semi-evisceration weight (SEW), evisceration weight (EW), breast muscle weight (BMW), leg muscle weight (LMW), leg weight (LW), abdominal fat weight (AFW), skin fat thickness (SFT), fat bandwidth (FBW) and some corresponding percentage traits. Meat quality traits included leg muscle fiber diameter (LFD), breast muscle fiber diameter (BFD), and intramuscular fat (IMF) of breast muscle. Furthermore, 1193 birds from 3 broiler breeds (Arbor Aceres, Hubbard, and Cobb), 7 native varieties (Dongxiang, Guifei, Gushi, Lushi, Blackbone, Wuhei and Xichuan), and 3 layer varieties (Hyline, Rhode island red and Lohmann) were used to assess the allele frequency of SNP in different breeds.

Forty two female Gushi chicken at 43 weeks old were obtained from Gushi Chicken Breeding Farm. All birds received ad libitum access to water and a standard commercial diet, which contained 2650 kcal kg-1 metabolic energy and 16% crude protein. Blood samples were drawn from the wing vein, and 11 tissues including hypothalamus, pituitary, ovary, liver, abdominal fat, pectoralis, subcutaneous fat, pancreas, duodenum, jejunum and kidney were collected after the birds were humanely slaughtered. The tissues samples were immediately snap-frozen in liquid nitrogen, and stored at -80 °C until use. The abdominal fat weight (AFW) and abdominal fat percentage (AFP = AFW / live body weight) were determined, liver lipid contents (LLC = Lipid drops area / Total area) were measured by oil red O staining analysis, and IMF (pectoral TG content) was measured by Tissue TG ELISA kits (Nanjing Jiancheng Bioengineering Institute, Nanjing, China) following manufacturer’s introductions. In addition, the additional samples of abdominal fat, liver and pectoralis were fixed for 48 h in 4% paraformaldehyde solution (Beijing Solarbio Science and Technology Co., Ltd., Beijing, China) for histological analysis.

To investigate whether *ELOVLs* expression level was affected by estrogen, the estrogen treatment and ovariectomy in vivo experiments were designed, respectively. In estrogen treatment experiment, twenty 10-week-old pullets in total were divided randomly into two groups with 10 birds in each group. Basing on our previous study (Li et al., 2020), the birds in one group was intramuscularly injected with 1.0 mg/kg of body weight of 17 β-estradiol (Sigma, St. Louis, MO, USA) which was dissolved in olive oil. The another group was served as a control, only injected with the same amount of olive oil. The birds were slaughtered to collect liver and hypothalamus tissues after treatment for 12 h. In ovariectomy experiment, 40 female chickens at 4 weeks old were divided randomly into two groups with 20 birds in every group. The birds in one group had their ovary removed, and the birds in another sham operation group were saved as a control. All of the 40 birds were raised up to 10 weeks of age. Ten healthy birds were slaughtered from each group to collect liver and hypothalamus tissues. The sampling and storing procedures were the same to that mentioned above.

### Association study between SNPs in *ELOVL* genes and growth, carcass and meat quality traits

The SNPs within the *ELOVL* genes were obtained by double-digest genotyping-by-sequencing (ddGBS) data of Gushi × Anka F2 chicken population [2]. The SNPs genotyping data were used for association analysis with growth, carcass and meat quality traits, and SNPs with significant effects on traits were further confirmed in 734 individuals of Gushi-Anka F2 chicken population by using Kompetitive Allele Specific PCR (KASP) [44]. Linkage disequilibrium analysis of the significantly associated SNPs was performed via haploview.

### Estrogen response elements prediction in promoter region of chicken *ELOVL* genes

The promoter sequence (2 kb) upstream from the transcription start site of *ELOVL* genes were extracted from UCSC database (http://genome.ucsc.edu/cgi-bin/hgGateway). The vertebrate estrogen response elements including EREs (ERα: MA0112.2, ERβ: MA0258.1) and AP1 (MA0099.2) element matrixes were downloaded from JASPAR (http://jaspar.genereg.net/). Then, the element matrixes and promoter sequence were submitted to MEME FIMO (http://meme-suite.org/tools/fimo) to scan for individual matches to each binding elements.

### Total RNA was extracted from the collected tissues using TRIzol reagent

Total RNA was extracted from the collected tissues using TRIzol reagent (Invitrogen, USA). First-strand cDNA was synthesized using a HiScript III RT SuperMix with gDNA wiper (Vazyme Biotech Co., Ltd) according to the manufacturer’s protocol. The expression levels of chicken *ELOVL*s and other genes were detected using quantitative real-time PCR (qRT-PCR). The specific primers were designed by NCBI Primer-BLAST (https://www.ncbi.nlm.nih.gov/tools/primerblast/) and synthesized by Sangon Biotech Co. Ltd. (Shanghai, China) (Additional file 9: Table S7). All reactions were carried out in triplicate. The relative level of each mRNA was normalized to the housekeeping gene *β-actin* and analysed by using the 1000 × 2^−Δct^ method.

### Histological analysis

The paraformaldehyde-fixed abdominal fat were sectioned and stained with hematoxylin–eosin (H&E), and the paraformaldehyde-fixed liver and pectoral tissues were stained with oil red O (Zhengzhou Nuohao Science and Technology Co., Ltd., Zhengzhou, China). Lipid drops area / Total area (%) was calculated to estimate the liver lipid content (%).

### Lipid molecules in pectoralis

We obtained the relative contents of different types of lipid molecules in pectorals of 43-week-old Gushi hens (20 birds) by using non-targeted lipidomic sequencing. A total of 733 lipid molecules of four types were detected in the pectoral tissue, including sterol lipids, sphingolipids, glycerophospholipids and glycerides. All lipid molecules of all 20 individuals were analyzed for correlation with *ELOVL3* mRNA expression in pectorals. The correlation and significance between *ELOVL*s expression and the prominently related lipid molecules were visualized by Cytoscape_v3.7.1.

### Statistical analysis

The association between SNPs and growth, carcass and meat quality traits was determined using the generalized linear mixed model (GLM) included with SPSS 23.0 (IBM, Chicago, IL, United States). The models used were as follows:

1 $${Y}_{iklm}=\upmu + {\mathrm{G}}_{i}+ {\mathrm{H}}_{k}+ {\mathrm{f}}_{l}+{\mathrm{e}}_{iklm}$$

2 $${\mathrm{Y}}_{iklm}=\upmu + {\mathrm{G}}_{i}+ {\mathrm{H}}_{k}+ {\mathrm{f}}_{l}+\mathrm{ b }\left({\mathrm{W}}_{iklm}-\overline{\mathrm{W} }\right)+ {\mathrm{e}}_{iklm}$$

Model I was used for SNP associations with growth and meat quality traits, whereas based on the effect of the carcass weight on carcass traits, Model II with carcass weight as a covariate was used for SNP associations with carcass traits. In these models, Y_*iklm*_ was the dependent variable (phenotypic value), μ was the observation mean and e_*iklm*_ is the random error, G_*i*_ was the fixed effect of genotype (*i* = genotypes), H_*k*_ was the fixed effect of hatching (*k* = 1, 2), f_*l*_ was the fixed effect of family (*l* = 1, 7), b was the regression coefficient for the carcass weight, W_*iklm*_ was the individual slaughter weight, and $\overline{{\text{W}}}$ was the average slaughter weight [42]. Significant differences between least squares means of the different genotypes were calculated using the least significant difference (LSD).

Statistical significance of the comparison between the two groups was examined by an independent-samples t-test, and statistical analysis among more than two groups was performed by one-way ANOVA. The results were presented as the mean ± SEM. The correlation between the gene expression and phenotypes was analyzed using Pearson correlation. *P < 0.05 was considered to be statistically significant, and **P < 0.01 was considered to be extremely significant.

## Supplementary Information

**Additional file 1: Table S1.** Association analysis of 51 SNPs in the *ELOVL *gene family with growth, carcass and meat quality traits in Gushi ×Anka F2 chickens.

**Additional file 2: Table S2.** Effects of 14 candidate SNPs on growth, carcass and meat quality traits.

**Additional file 3: Figure S1.** Linkage disequilibrium analysis (LD) of SNPs in ELOVL genes in Gushi-Anka F2 population.

**Additional file 4: Table S3. **Putative ERE and AP1 element in chicken ELOVL genes promoter.

**Additional file 5: Figure S2.** The expression levels of ELOVL genes in hypothalamus, liver, abdominal fat and pectorals of AFL and AFH.

**Additional file 6: Table S4.** List of lipids in intramuscular fat correlated with ELOVL3 expression in pectorals.

**Additional file 7:** **Table S5.** Data sources for red jungle fowl and layers used in this study.

**Additional file 8: Table S6.** All genomic SNPs in gene body and 2 kb promoter of ELOVL gene family used for PCA analysis.

**Additional file 9: Table S7.** Gene specific primers used for qRT-PCR.

## Data Availability

The whole genome SNPs information of *ELOVL* gene family among breeds are included in Additional files of this study. And the other original data that support those study findings can be obtained by contacting the first author if required.

## Abbreviations

LCFA: Long chain fatty acids
VLC-SFA: Very long chain saturated fatty acids
SNPs: Single nucleotide polymorphisms
PCA: Principal component analysis
AFW: Abdominal fat weight
AFP: Abdominal fat percentage
IMF: Intramuscular fat contents
LLC: Liver lipid contents
KASP: Kompetitive Allele Specific PCR
