# Supplementary material for: ELOVL gene family plays a virtual role in response to breeding selection and lipid deposition in different tissues in chicken (Gallus gallus)
Source: BMC Genomics. 2022 Oct 17;23:705. doi: 10.1186/s12864-022-08932-8 (PMC9575239; doi:10.1186/s12864-022-08932-8)
Supplement: Supplementary file 3 — Additional file 3: Figure S1. Linkage disequilibrium analysis (LD) of SNPs in ELOVL genes in Gushi-Anka F2 population. [file 12864_2022_8932_MOESM3_ESM.docx]

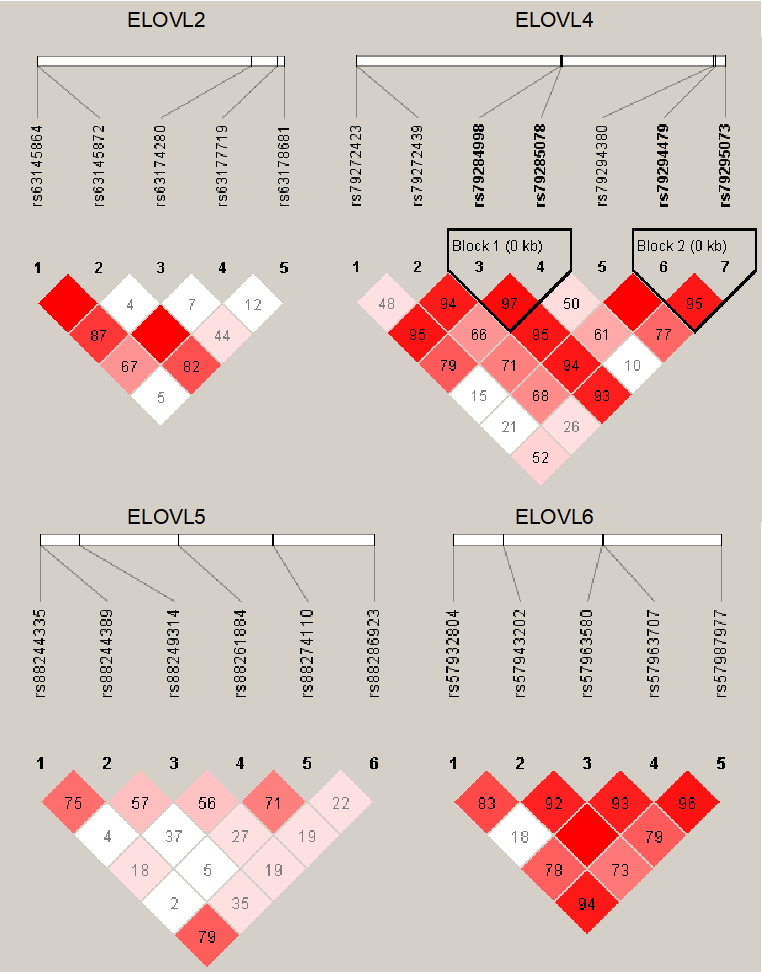


**Figure S1.** Linkage disequilibrium analysis (LD) of SNPs in *ELOVL* genes in Gushi × Anka F2 population. LD was carried out by Haploview. The pairwise LD coefficient were evaluated by the D’ value.
