# Supplementary material for: ELOVL gene family plays a virtual role in response to breeding selection and lipid deposition in different tissues in chicken (Gallus gallus)
Source: BMC Genomics. 2022 Oct 17;23:705. doi: 10.1186/s12864-022-08932-8 (PMC9575239; doi:10.1186/s12864-022-08932-8)
Supplement: Supplementary file 5 — Additional file 5: Figure S2. The expression levels of ELOVL genes in hypothalamus, liver, abdominal fat and pectorals of AFL and AFH. [file 12864_2022_8932_MOESM5_ESM.docx]

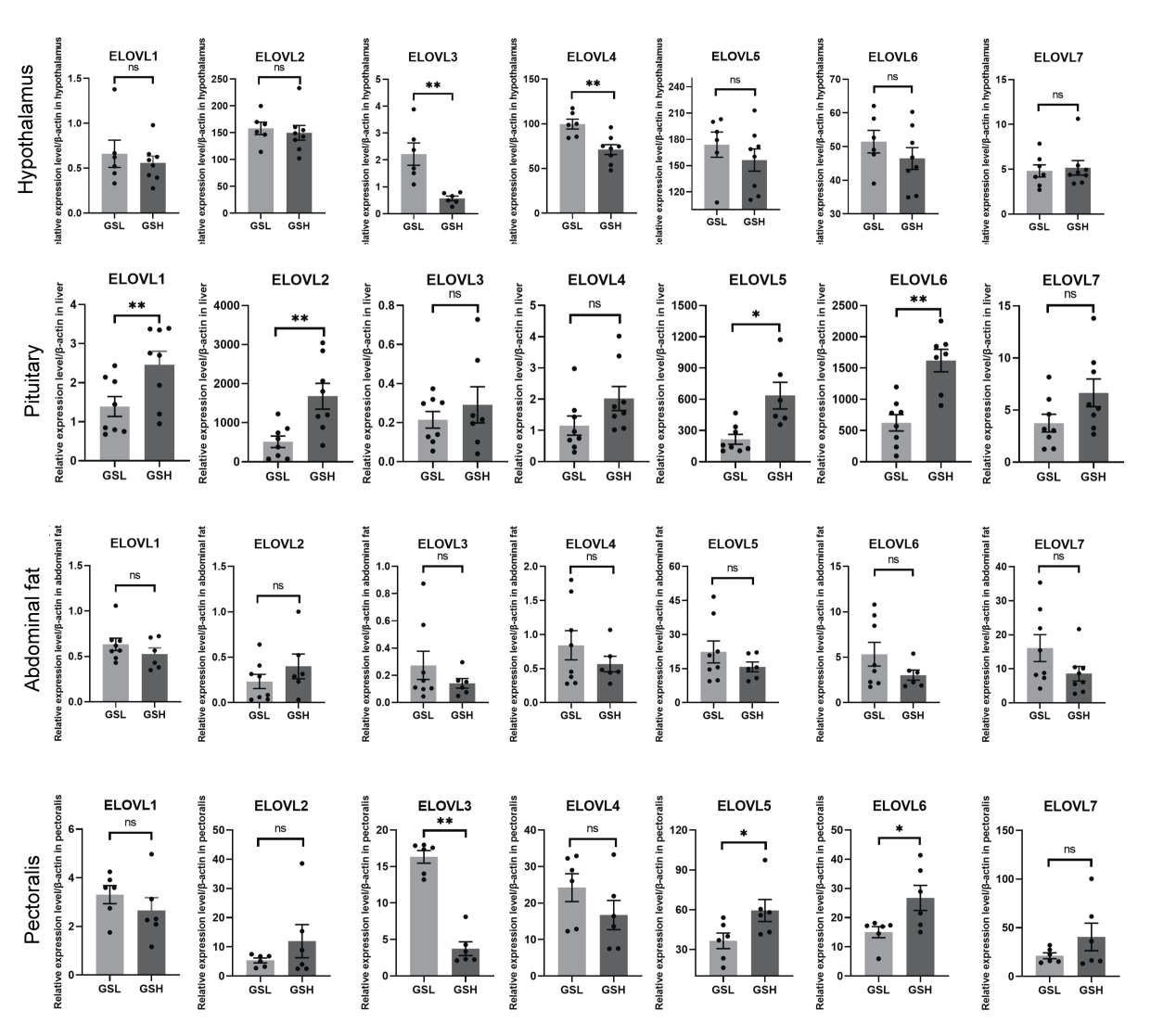


**Figure S2.** The expression levels of *ELOVL* genes in hypothalamus, liver, abdominal fat and pectorals of AFL and AFH. AFL, the low abdominal fat group of Gushi chicken. AFH, the high abdominal fat group of Gushi chicken, The mRNA levels of genes were normalized to *β-actin*. Results are presented as the mean ± SEM and the normalized expression values for all individuals (n = 6-8). Significant difference: * *p* < 0.05, ** *p* < 0.01. ns: no difference.
